# Obstetric anal sphincter injuries (OASIS): using transperineal ultrasound (TPUS) for detecting, visualizing and monitoring the healing process

**DOI:** 10.1186/s12905-022-01915-7

**Published:** 2022-08-10

**Authors:** Anna-Lena Stickelmann, Lieven Nils Kennes, Miriam Hölscher, Charlotte Graef, Tomas Kupec, Julia Wittenborn, Elmar Stickeler, Laila Najjari

**Affiliations:** 1 Gynaecology and Obstetrics, Uniklinik RWTH Aachen, Aachen, Germany; 2 Department of Economics and Business Administration, Hochschule Stralsund, Stralsund, Germany

**Keywords:** Transperineal ultrasound, Obstetric anal sphincter injury, Third-degree perineal tear, Anal sphincter injuries

## Abstract

**Purpose:**

The aim of this study was to examine whether OASIS, and its extent, can be confirmed or excluded using transperineal ultrasound (TPUS). A further objective of this study was to monitor the healing process over a period of 6 months and to establish a connection between the sonographic appearance of obstetric anal sphincter injury (OASIS) and anal incontinence.

**Materials and methods:**

In this retrospective clinical study, women with OASIS who gave birth between March 2014 and August 2019 were enrolled. All the patients underwent TPUS 3 days and 6 months after delivery. A GE E8 Voluson ultrasound system with a 3.5–5 MHz ultrasound probe was used. The ultrasound images showed a third-degree injury, with the measurement of the width of the tear and its extent (superficial, partial, complete, EAS and IAS involvement). A positive contraction effect, a sign of sufficient contraction, was documented. Six months after delivery, a sonographic assessment of the healing (healed, scar or still fully present) was performed. A Wexner score was obtained from each patient. The patients’ medical histories, including age, parity, episiotomy and child’s weight, were added.

**Results:**

Thirty-one of the 55 recruited patients were included in the statistical evaluation. Three patients were excluded from the statistical evaluation because OASIS was excluded on TPUS 3 days after delivery. One patient underwent revision surgery for anal incontinence and an inadequately repaired anal sphincter injury, as shown sonographic assessment, 9 days after delivery. Twenty patients were excluded for other reasons. The results suggest that a tear that appears smaller (in mm) after 3 days implies better healing after 6 months. This effect was statistically significant, with a significance level of alpha = 5% (*p* = 0.0328). Regarding anal incontinence, women who received an episiotomy had fewer anal incontinence symptoms after 6 months. The effect of episiotomy was statistically significant, with a significance level of alpha = 5% (*p* = 0.0367).

**Conclusion:**

TPUS is an accessible, non-invasive method for detecting, quantifying, following-up and monitoring OASIS in patients with third-degree perineal tears. The width, as obtained by sonography, is important with regard to the healing of OASIS. A mediolateral episiotomy seems to prevent anal incontinence after 6 months.

## Introduction

In Germany, OASIS is rare. In particular, a third-degree injury of the anal sphincter occurs in 1.74% of women who have a vaginal birth [1]. There is a risk of 30–60% that OASIS will lead to anal incontinence [2], which affects the patient’s quality of life. If symptoms, such as flatulence and loss of liquid or even solid stools, persist there may be a medical indication for revision surgery. An inadequate sphincter repair may increase the risk of exacerbated symptoms after a subsequent vaginal delivery [3]. The incidence of wound dehiscence after primary repair of OASIS is 6.9% [4]. There is solid evidence for performing early revision surgery within 14 days instead of delayed repair after 3–6 months [5]. This leads to the question of how to monitor women after an injury of the anal sphincter.

The true extent of an obstetric anal sphincter injury (OASIS) is difficult to assess in clinical examination immediately after delivery because of swelling and bleeding. Therefore, the German Guideline recommends soliciting the expertise of an experienced examiner for cases with unclear wound conditions [1]. Zetterström et al. [6] pointed out that OASIS is often inadequately diagnosed and that primary repair frequently results in persisting defects. The aim of this study was to examine whether OASIS and its extent can be confirmed or excluded using transperineal ultrasound (TPUS). A further objective of this study was to monitor the healing process over a period of 6 months and to establish a connection between the sonographic appearance of the perineal tear and the presence of anal incontinence. In the case of an inadequate repair, this procedure may create the opportunity for contemporary revision surgery.

Endoanal ultrasound (EAUS) is still considered to be the “gold standard” for visualizing the anal sphincter [7]. In a previous study, our study group was able to show that TPUS is a reproducible, pain-free and easily applied method to visualize the anal canal [8]. It is less invasive, less expensive and readily available compared to EAUS [9]. Huang et al. and Lee et al. showed that the anal sphincter can be depicted in TPUS and that the measurement of the external anal sphincter (EAS) and the internal anal sphincter (IAS) is reproducible [10, 11]. In addition, Lee et al. showed interobserver reliability in detecting sphincter defects by TPUS [12] after delivery. Moreover, Valsky et al. found 7.9% occult sphincter defects with TPUS [13].

**Fig. 1:**
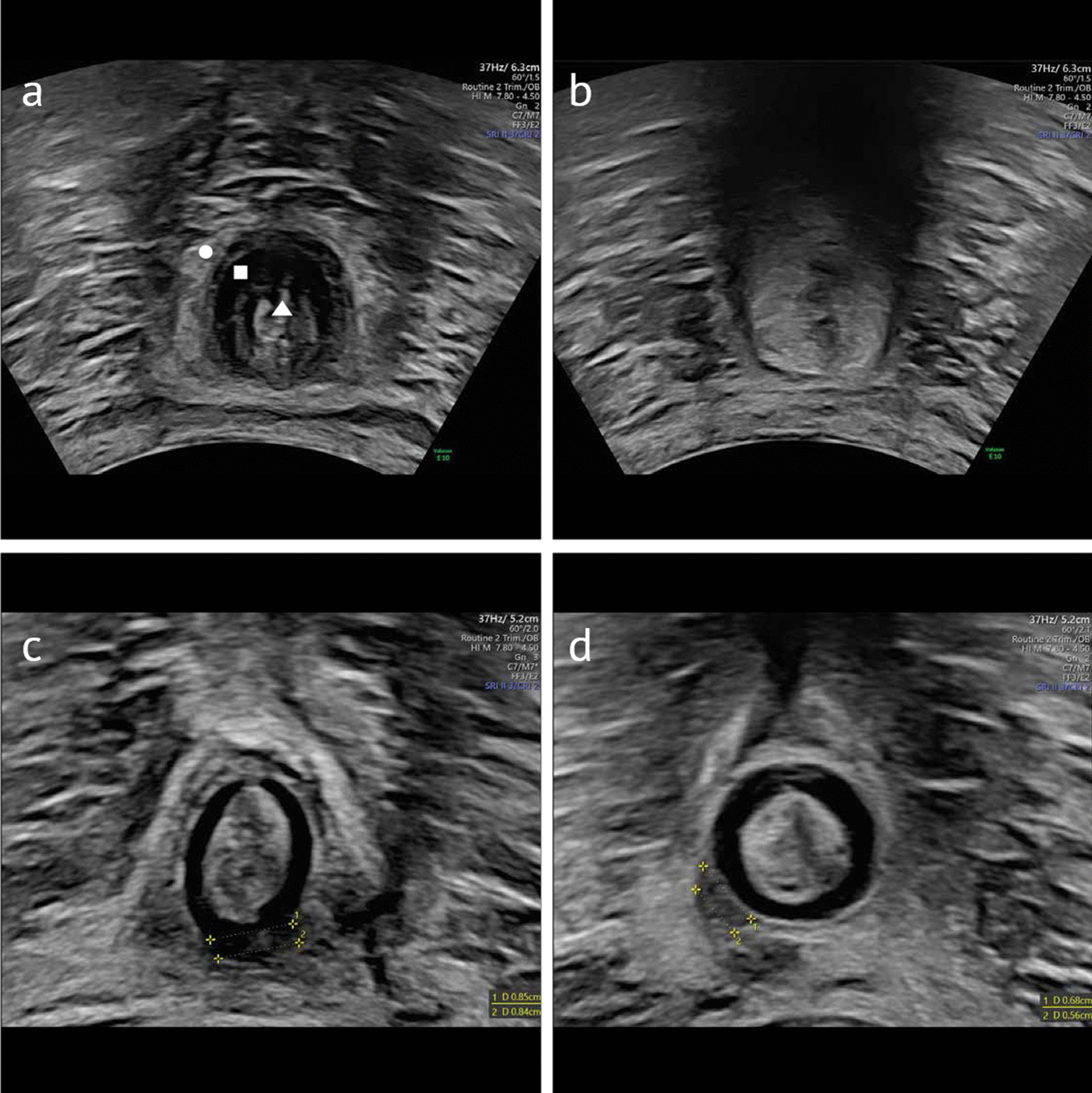
Transperineal ultrasound of the anal sphincter. **a** Intact anal sphincter EAS IAS lumen **b** positive contraction effect—the sphincter disappears **c** complete tear of the EAS 3 days after delivery **d** scar of the EAS after 6 months

When examining the anal sphincter by TPUS, four layers can be viewed (from the outer to the inner part): the EAS as a hyperechogenic mass, the hypoechogenic IAS, followed by the mucous membrane and finally the lumen of the anal canal (Fig. 1a). All layers can be depicted in a sagittal and a transversal cutting plane.

Even after sphincter repair, it is possible to visualize the injury. According to the World Health Organization’s generally accepted classification of third-degree lacerations, including class III-a (less than 50% of EAS), III-b (more than 50% of EAS) and III-c (EAS and IAS involvement) [3], the extent of the laceration can also be shown by TPUS.

An episiotomy is performed to aid a difficult delivery. Episiotomy is intended to prevent obstetric anal sphincter injuries (OASIS) and accelerate delivery in the case of foetal distress. A 2017 Cochrane analysis showed that women who selectively underwent episiotomy had less severe perineal trauma [14]. Nevertheless, according to the German guidelines, a median episiotomy is accompanied by a higher risk of OASIS, and a mediolateral episiotomy should be used restrictively [1].

Birthing positions such as squatting, lithotomy and the spinal position are known risk factors of OASIS [1, 15], whereby perineal techniques such as warm compresses and massage seem to prevent OASIS [1, 16].

Ten percent of women who have a vaginal birth suffer from anal incontinence after delivery [17]. Operative vaginal delivery, OASIS and prolonged labour are known risk factors of faecal incontinence [18]. The Wexner Scale is an instrument for scoring the severity of anal incontinence; the scale assesses symptoms from the past 4 weeks, including loss of liquid or solid stools, flatulence, sanitary napkin use and lifestyle alterations. The maximum number of points in this score is 20, which means complete incontinence. Anal incontinence has a major impact on quality of life; its presence may compromise social and intimate relationships, employment status and self-esteem [17]. In conclusion, anal sphincter function is only one of many factors affecting continence or the lack thereof [17].

## Material and methods

### Study design

In this retrospective clinical study, women with OASIS who had given birth in the RWTH University Clinic in Aachen between March 2014 and August 2019 were enrolled. All patients underwent an examination via TPUS 3 days and 6 months after delivery. The standardized examination was performed in the urogynaecological department by an experienced, AGUB (Arbeitsgemeinschaft für Urogynäkologie und plastische Beckenbodenrekonstruktion e.V.)- II certified examiner. The tears were measured using the volumes on the stored 4D-ultrasound. The clinical data of the patients were collected and pseudoanonymized. The OASIS was sutured according to the German guidelines with 3–0 thread thickness, atraumatic sutures and end-to-end anastomosis of the external and internal anal sphincters [1].

### TPUS

During the TPUS examination, the ultrasound probe was placed on the anal opening of the patient and tilted 10°–20° in the ventral direction to locate the anal sphincter. Figure 1a shows an intact anal sphincter.

A GE E8 Voluson ultrasound system with a 3.5–5 MHz ultrasound probe was used. All patients were instructed to perform a contraction manoeuvre “relax-squeeze-relax”. 4D-ultrasound volumes were acquired in the GE E8 Voluson ultrasound system. The measurements were taken with GE Healthcare 4D View software. Tomographic ultrasound imaging (TUI) was used to find the best possible section. Analogous to the previous study “Perianal ultrasound (PAUS): visualization of sphincter muscles and comparison with digital-rectal examination (DRE) in females”, the TUI slices were chosen [8]. The lowest slice was defined as the slice in which the external anal sphincter muscle (EAS) and internal anal sphincter muscle (IAS) were completely seen for the first time, starting from the anocutaneous transition zone and moving in the cranial direction. In the cranial direction, four slices with a 2 mm interslice distance were set. These four TUI slices cranial of the lowermost slice were evaluated. At these slice levels, the contraction manoeuvre could be performed without losing the plane dynamically. The slice that showed the OASIS most exactly and to the greatest extent was chosen for the measurements. The quality requirement was that both sphincter muscles were completely observed in the plane.

In case an injury of the EAS or IAS could not be visualized, the complete 4D-ultrasound volume was explored to exclude OASIS.

### Variables

The healing and continence statuses after 6 months were identified as primary endpoints:

For the healing status, sonography was performed to assess healing after 6 months. The defects were divided into the following categories: healed, scar or still fully present. Figure 1c shows a complete defect of the EAS after 3 days; in addition, Fig. 1d shows a scar at the 6 months follow-up examination. For statistical analyses, the endpoint was dichotomized, “healed” versus “scar” or “still fully present”.

For the continence status, the Wexner Score for anal incontinence was obtained for all the patients at the time of both visits. For statistical analyses, the Wexner Score was dichotomized according to whether stool incontinence was present (a Wexner Score higher than 0) or absent (a Wexner Score equal to zero).

Furthermore, the following assessments were made:

The ultrasound images showed a third-degree injury, the width and the extent of the tear (superficial, partial, complete, EAS and IAS involvement). Due to the low sample size (*n* = 31) and only observing one subject in “EAS and IAS” and one subject in “superficial”, the extent of the lesion was pooled into two categories: (1) moderate (including “superficial” and “partial” tears) and (2) severe (including “complete” and “EAS and IAS”).

**Fig. 2:**
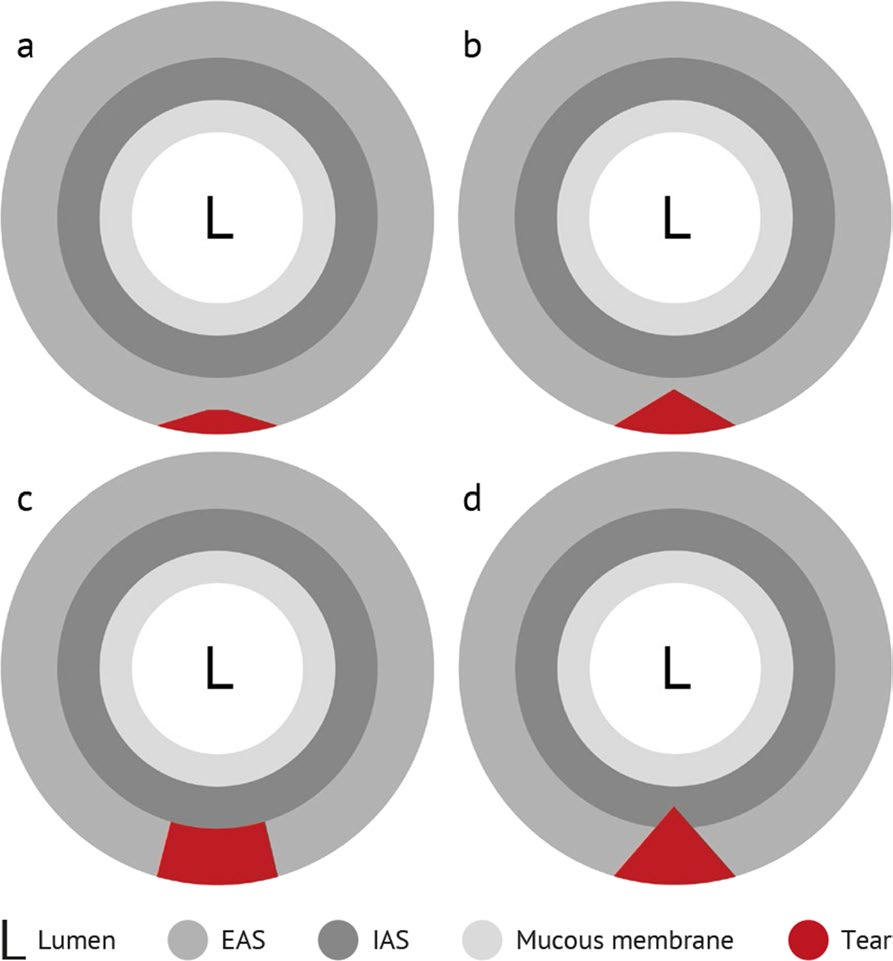
Model of a third-degree OASIS. **a** superficial tear of the EAS **b** partial tear of the EAS **c** complete tear of the EAS **d** EAS and IAS affected

Figure 2 shows a model of a third-degree OASIS. Figure 1c shows the measurement of the width.

Furthermore, we documented a positive contraction effect if the sphincter disappeared from the sonographic window during the manoeuvre, which indicated sufficient contraction. A positive contraction effect is shown in Fig. 1b.

We added data from the patients’ medical histories, including age, parity, presence of episiotomy and child’s weight.

### Statistics

Continuous variables are expressed as the mean values ± standard deviation (SD). Categorical data are represented by absolute frequencies and/or percentages. involvement).

To investigate both dichotomized primary endpoints (healing and continence status), logistic regression was conducted to investigate the influence of the width of the tear, the extent of the tear, the contraction status, the continence status and episiotomy on the respective endpoints. Model selection was performed using the AIC criteria. The best-fitting model, with the lowest AIC, is reported in the Results section.

All tests were two-sided and were assessed at the 5% significance level. Because of the exploratory nature of the study, the significance level was not adjusted to account for multiplicity. All statistical analyses were conducted using R [19] statistical software.

## Results

Between March 2014 and August 2019, 4396 women had vaginal births in the RWTH University Clinic in Aachen. In the clinical examination, 1,25 percent (*n* = 55) of the patients had a third-degree injury.

**Fig. 3:**
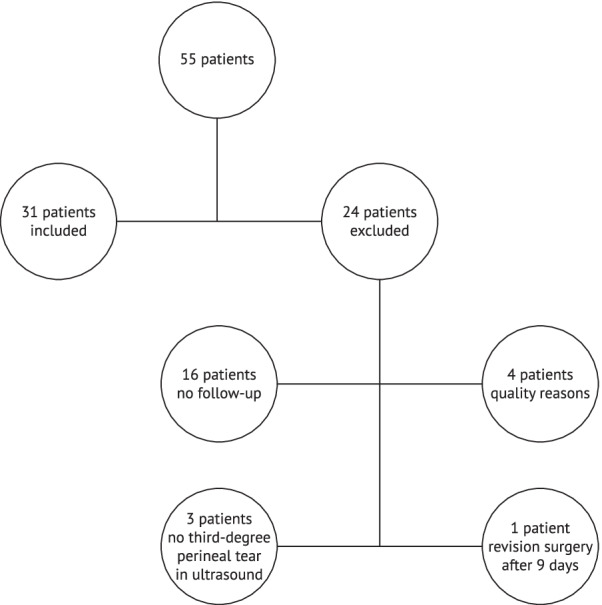
Cohort

Twenty-four patients were excluded from the statistical evaluation for the following reasons: 16 patients did not appear for the 6 months follow-up examination, 4 patients had ultrasound images that were unable to be analysed for quality reasons, 3 patients showed no third-degree sphincter injury in the sonographic assessment and 1 patient underwent revision surgery 9 days after delivery. The data of 31 patients were entered into the statistical evaluation. (Fig. 3).

Most of the patients (93.55%) had their first vaginal delivery. They were aged between 23 and 38 years. A total of 35.48% of the patients underwent an operative delivery. Another 35.48% underwent a mediolateral episiotomy. A total of 16.13% of the newborns had a birthweight of more than 4000 g, and the average child’s weight was 3569 g ± 489.34 g. In general, the average child’s birthweight in Germany in 2017 was 3480 g [20]. Shoulder dystocia occurred in 9.68% of the deliveries in the cohort. (Table 1).

**Table 1:** Baseline Characteristics

Characteristic	*n*|%
First vaginal delivery	29|93.55
Instrumental vaginal delivery	11|35.48
Episiotomy (all mediolateral)	11|35.48
Foetal macrosomia (> = 4000 g)	5|16.13
Shoulder dystocia	3|9.68
	Mean|SD
Child’s weight (g)	3568.55|489.34

In the sonographic assessment performed 3 days after delivery, superficial involvement of the EAS was observed in one patient and the EAS and the IAS were both affected in one patient. After categorizing the lesions into “moderate” and “severe” lesions, 19% of the lesions were moderate and 81% were severe. (Table 2).

**Table 2:** Categorization of the type of lesion into two groups

Type of lesion	*n*|%	New category	*n*|%
Superficial	1|3		
Partial	5|16	Moderate	6|19
Complete	24|77		
EAS and IAS involvement	1|3	Severe	25|81

Regarding the width of the perineal tear in all 31 patients, the mean width decreased after 6 months, regardless of the type of lesion. The decrease in the width after 6 months was greater in the patients with severe lesions than in the patients with moderate lesions. (Table 3).

**Table 3:** Development of the lesions within 6 months

Characteristic	3 days		6 months		Difference	
	Mean	SD	Mean	SD	Mean	SD
Mean width overall (cm)	0.564	0.378	0.273	0.182	− 0.291	0.297
Severe lesions (cm)	0.592	0.384	0.279	0.174	− 0.312	0.314
Moderate lesions (cm)	0.448	0.359	0.247	0.231	− 0.202	0.205
Episiotomy (cm)	0.51	0.355	0.30	0.246	− 0.21	0.270
No episiotomy (cm)	0.59	0.396	0.258	0.141	− 0.336	0.308

Considering the mean size of the defects depends on whether an episiotomy is performed, patients with an episiotomy appear to have a smaller mean defect size after 3 days but a larger mean defect size after 6 months in comparison to patients without episiotomy. (Table 3).

Three days after delivery, most patients had no contraction effect on ultrasound, but contraction effects were observed in all patients after 6 months. (Table 4) The incidence of anal incontinence symptoms decreased during the 6 months following delivery. (Table 5).

**Table 4:** Development of the contraction effect

	3 days (*n*|%)	6 months (*n*|%)
Contraction effect	3|9.68	31|100
No contraction effect	28|90.32	0|0

**Table 5:** Development of faecal incontinence

	3 days (*n*|%)	6 months (*n*|%)
Anal incontinence	14|45.16	10|32.26
No anal incontinence	17|54.84	21|67.74

Comparing the type of lesion 3 days and 6 months after delivery, smaller-degree lesions showed more significant healing. Patients already suffering from incontinence symptoms 3 days after giving birth (positive Wexner Score) had a lower chance of being categorized as healed after 6 months. Episiotomy had no effect on healing. (Table 6).

**Table 6:** Healing of the third-degree perineal tears

Characteristic	Lesion still present or scar	Healed
Overall (*n*|%)	8|25.8	23|74.2
Severe (*n*|%)	7|28.0	18|72.0
Moderate (*n*|%)	1|16.7	5|83.3
No episiotomy (*n*|%)	5|25	15|75
Episiotomy (*n*|%)	3|27	8|72.72
Anal incontinence after 3 days (*n*|%)	5|35.7	9|64.28
No anal incontinence after 3 days (*n*|%)	3|17.6	14|82.35
Mean width 3 days (cm)	0.398 (± 0.256)	0.643 (± 0.406)
Mean width 6 months (cm)	0.207 (± 0.108)	0.304 (± 0.203)

Only 32% of the patients had anal incontinence symptoms after 6 months. Women with moderate lesions had fewer symptoms after 6 months than women with severe lesions. Women with an episiotomy had fewer anal incontinence symptoms. Paradoxically, women with larger defects in the sonographic assessment had fewer anal incontinence symptoms after 6 months (Table 7). There might be a correlation because women with episiotomy had larger defects after 6 months when compared to women without episiotomy (Table 3).

**Table 7:** Anal incontinence after 6 months

Characteristic	Stool incontinence after 6 months	No stool incontinence after 6 months
Overall (*n*|%)	10|32	21|68
Severe (*n*|%)	9|36	16|64
Moderate (*n*|%)	1|16.7	5|83.3
Episiotomy (*n*|%)	1|9	10|91
No episiotomy (*n*|%)	9|45	11|55
Mean width 3 days (cm)	0.398 (± 0.256)	0.643 (± 0.406)
Mean width 6 months (cm)	0.207 (± 0.108)	0.304 (± 0.203)

A multivariate analysis of healing after 6 months, as primary endpoint, demonstrates the importance of the width after 3 days, the contraction effect and the Wexner Score after 3 days. All three variables remained in the final, best-fitting model. The negative coefficients indicate that the healing probability decreases as parameter values increase. While the negative coefficients of the width after 3 days and the stool incontinence (0/1) make sense from a medical perspective, the negative coefficient of the contraction effect might be a statistical artefact. Only 3 patients had a positive contraction effect after 3 days, of which just one had a favourable healing status, possibly due to other reasons. Therefore, a sensitivity analysis was performed, in which the contraction effect was locally removed in the final statistical model. The results of the main and sensitivity analyses are shown in Table 8.

**Table 8:** Primary endpoint healing

	Model1		Model 2	
	Coefficient	*p*-value	Coefficient	*p*-value
Width 3 days	− 0.3532	0.0279	− 0.2987	0.0328
Anal incontinence 3 days	− 1.8272	0.1256	− 1.7184	0.1078
Contraction effect	− 2.3220	0.0961		

**Fig. 4:**
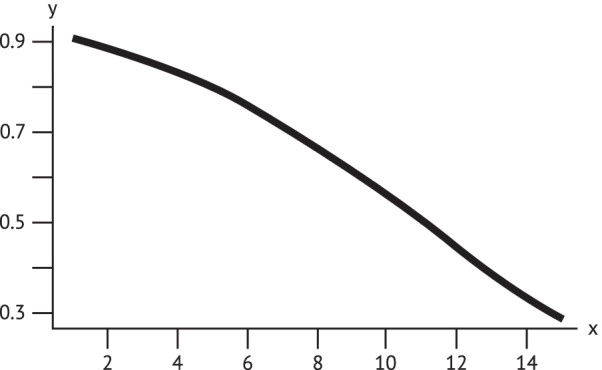
Probability of healing. **x**: tear in mm after 3 days; **y**: probability of healing after 6 months

The results show a smaller tear (in mm) after 3 days, has better healing after 6 months (Fig. 4). This effect was statistically significant, at a significance level of alpha = 5% (*p* = 0.0328). Women who already had anal incontinence symptoms after 3 days had poorer chances of healing after 6 months, but no statistical signficant effect was identified.

In the second multivariate analysis, the width of the tear after 3 days and the presence of episiotomy remained the best-fitting model, with a Wexner score after 6 months as primary endpoint. (Table 9).

**Table 9:** Primary endpoint faecal continence

	Coefficient	*p*-value
Width 3 days	− 2.9012	0.0630
Episiotomy	− 2.5653	0.0367

Women with an episiotomy had fewer anal incontinence symptoms after 6 months. The effect of episiotomy was statistically significant, at a significance level of alpha = 5% (*p* = 0.0367). Paradoxically, women with wider tears after 3 days also had fewer incontinence symptoms after 6 months; however, this effect was not statistically significant.

## Discussion

Anal incontinence is a serious issue, and therapeutic options are limited [21]. Delivery is an important risk factor for anal incontinence, particularly after a third- or fourth-degree perineal tear [17, 18]. The risk of reoccurrence at the next delivery is 4–8% [3]. After successful repair, a subsequent vaginal delivery is justifiable [3]. Barbosa et al. [22] showed that early secondary repair, within 21 days, has similar long-term outcomes as late sphincter repair. Harvey et al. [3] pointed out that a persistent defect of the EAS may increase the risk of exacerbated symptoms after a subsequent delivery. Early sonographic detection of an inadequately repaired birth injury could improve the management of OASIS.

TPUS is a suitable method for examining OASIS because it is reproducible [10, 11], readily available, painless and provides the opportunity for a follow-up.

This study could show that performing a TPUS 3 days after delivery is reasonable for patients with OASIS. This procedure can either exclude a higher degree of birth injury or confirm adequate sphincter repair. Furthermore, the extent of OASIS can be quantified. The woman can be assured that she will certainly not suffer from anal incontinence due to OASIS. In the event of an inadequate result, the examination on the third day after delivery provides the opportunity for early revision surgery or a conservative approach, such as intensified pelvic floor exercise, and a follow-up examination after 6 months.

In addition, by monitoring the healing process and performing a follow-up examination after 6 months, physicians and patients become more aware of pelvic floor problems and anal incontinence. Harvey et al. recommend to “disclose to women the degree of injury and arrange a follow-up” [3] and demand “detailed documentation of the injury and its repair” [3]. TPUS can ensure that the injury is healed. Furthermore, the examination provides an opportunity to prioritize the patients’ concerns and resolve important questions related to OASIS: Will the patient presumably suffer from anal incontinence at an advanced age? Will OASIS have consequences in future deliveries?

The results show that, with the natural healing process, it is very probable that the width of third-degree tears will decrease during the 6 months after delivery. Furthermore, the number of patients with anal incontinence symptoms declines. This is an argument that supports a “wait and watch” approach for at least 6 months to assess symptom severity in hopes of avoiding an unnecessary surgery.

All the patients regained a contraction effect after 6 months, regardless of the severity of OASIS or the width of the tear. This indicates that other factors, such as pelvic floor function, might affect the quality of the contraction. In view of the statistical impact of the contraction effect on healing, the multivariate analysis showed that the probability of healing decreases with a positive contraction effect. This result does not make sense from a medical perspective. A similar distribution in a larger sample size is questionable. Additionally, the explanation could be that the contraction effect is possibly influenced by pelvic floor function and OASIS.

This study showed that reductions in the width of the tear (in mm) improves healing. This stresses the importance of an adequate sphincter repair as well as the importance of preventing OASIS, such as increasing focus on the maternal birth position and protecting the perineum by the midwife.

As patients with episiotomy had fewer anal incontinence symptoms after 6 months, thus episiotomy seems to prevent anal incontinence. Additionally, the width of the tear after 3 days was smaller. However, the mean width of the tear after 6 months in patients with episiotomy was larger.

We assume that sphincter repairs as a result of previous episiotomies are easier because the tear of the perineum is controlled. This could explain the smaller sonographic appearance of the tear after 3 days, possibly also due to less swelling. After 6 months, patients with episiotomy had fewer anal incontinence symptoms despite larger tears because the whole pelvic floor was protected by performing the episiotomy.

Due to the small sample size, we were unable to determine an exact cut-off for the width of the perineal tear 3 days after delivery that would allow a decision for or against revision surgery. The clinical symptoms and the surgeon’s experience therefore have to be included in the decision-making. Further studies with a larger cohort need to be performed to determine a cut-off.

## Conclusion

TPUS is a readily available, non-invasive method used for detecting, quantifying, following-up and monitoring third-degree perineal tears in patients with OASIS. The sonographic width is important for the healing of OASIS. A mediolateral episiotomy seems to prevent anal incontinence after 6 months.

## Data Availability

The datasets used and/or analysed during the current study are available from the corresponding author on reasonable request.

## References

[1] Deutsche Gesellschaft für Gynäkologie und Geburtshilfe. Leitlinie zum Management von Dammrissen III. und IV. Grades nach vaginaler Geburt. https://www.awmf.org/uploads/tx_szleitlinien/015-079l_S2k_Dammriss-III-IV-Grades_2020-12_1.pdf.

[2] Schumpelick V, Rath W, Willis S, Faridi A (2002). Anale Inkontinenz nach vaginaler Geburt: Ein Argument für den Kaiserschnitt auf Wunsch ?. Deutsches Ärzteblatt.

[3] Harvey M-A, Pierce M, Walter J-E, Chou Q, Diamond P, Epp A (2015). Obstetrical anal sphincter injuries (OASIS): prevention, recognition, and repair. J Obstet Gynaecol Can.

[4] Okeahialam NA, Wong KW, Thakar R, Sultan AH (2022). The incidence of wound complications following primary repair of obstetric anal sphincter injuries (OASIs): a systematic review and meta-analysis. Am J Obstet Gynecol.

[5] Okeahialam NA, Thakar R, Sultan AH (2021). Early secondary repair of obstetric anal sphincter injuries (OASIs): experience and a review of the literature. Int Urogynecol J.

[6] Zetterström J, López A, Holmström B, Nilsson BY, Tisell A, Anzén B, Mellgren A (2003). Obstetric sphincter tears and anal incontinence: an observational follow-up study. Acta Obstet Gynecol Scand.

[7] Ledgerwood-Lee M, Zifan A, Kunkel DC, Sah R, Mittal RK (2019). High-frequency ultrasound imaging of the anal sphincter muscles in normal subjects and patients with fecal incontinence. Neurogastroenterol Motil.

[8] Hölscher M, Gräf C, Stickelmann A-L, Stickeler E, Najjari L (2021). Perianal ultrasound (PAUS): visualization of sphincter muscles and comparison with digital-rectal examination (DRE) in females. BMC Womens Health.

[9] Albuquerque A, Pereira E (2016). Current applications of transperineal ultrasound in gastroenterology. World J Radiol.

[10] Huang W-C, Yang S-H, Yang J-M (2007). Three-dimensional transperineal sonographic characteristics of the anal sphincter complex in nulliparous women. Ultrasound Obstet Gynecol.

[11] Lee JH, Pretorius DH, Weinstein M, Guaderrama NM, Nager CW, Mittal RK (2007). Transperineal three-dimensional ultrasound in evaluating anal sphincter muscles. Ultrasound Obstet Gynecol.

[12] Peschers UM, DeLancey JO, Schaer GN, Schuessler B (1997). Exoanal ultrasound of the anal sphincter: normal anatomy and sphincter defects. Br J Obstet Gynaecol.

[13] Valsky DV, Messing B, Petkova R, Savchev S, Rosenak D, Hochner-Celnikier D, Yagel S (2007). Postpartum evaluation of the anal sphincter by transperineal three-dimensional ultrasound in primiparous women after vaginal delivery and following surgical repair of third-degree tears by the overlapping technique. Ultrasound Obstet Gynecol.

[14] Jiang H, Qian X, Carroli G, Garner P (2017). Selective versus routine use of episiotomy for vaginal birth. Cochrane Database Syst Rev.

[15] Huang J, Zang Y, Ren L-H, Li F-J, Lu H (2019). A review and comparison of common maternal positions during the second-stage of labor. Int J Nurs Sci.

[16] Aasheim V, Nilsen ABV, Reinar LM, Lukasse M (2017). Perineal techniques during the second stage of labour for reducing perineal trauma. Cochrane Database Syst Rev.

[17] Meyer I, Richter HE (2016). Evidence-based update on treatments of fecal incontinence in women. Obstet Gynecol Clin North Am.

[18] Swash M (1993). Faecal incontinence. BMJ.

[19] R Core Team. A language and environment for statistical computing. Vienna, Austria: R Foundation for Statistical Computing; 2018.

[20] Kiserud T, Piaggio G, Carroli G, Widmer M, Carvalho J, Neerup Jensen L (2017). The World Health Organization fetal growth charts: a multinational longitudinal study of ultrasound biometric measurements and estimated fetal weight. PLoS Med.

[21] Saldana Ruiz N, Kaiser AM (2017). Fecal incontinence - challenges and solutions. World J Gastroenterol.

[22] Barbosa M, Glavind-Kristensen M, Christensen P (2020). Early secondary repair of obstetric anal sphincter injury: postoperative complications, long-term functional outcomes, and impact on quality of life. Tech Coloproctol.

